# Determination of the Variability of Biophenols and Mineral Nutrients in Olive Leaves with Respect to Cultivar, Collection Period and Geographical Location for Their Targeted and Well-Timed Exploitation

**DOI:** 10.3390/plants9121667

**Published:** 2020-11-27

**Authors:** Igor Lukić, Igor Pasković, Paula Žurga, Valerija Majetić Germek, Mia Brkljača, Šime Marcelić, Dean Ban, Kristina Grozić, Marina Lukić, Zoran Užila, Smiljana Goreta Ban

**Affiliations:** 1 Institute of Agriculture and Tourism, Department of Agriculture and Nutrition, K. Huguesa 8, 52440 Poreč, Croatia; igor@iptpo.hr (I.L.); dean@iptpo.hr (D.B.); grozic@iptpo.hr (K.G.); marina@iptpo.hr (M.L.); zoran@iptpo.hr (Z.U.); smilja@iptpo.hr (S.G.B.); 2 Centre of Excellence for Biodiversity and Molecular Plant Breeding, Svetošimunska 25, 10000 Zagreb, Croatia; 3 Teaching Institute of Public Health of Primorsko-goranska County, Krešimirova 52a, 51000 Rijeka, Croatia; paula.zurga@zzjzpgz.hr; 4 Faculty of Medicine, Department of Food Technology and Control, University of Rijeka, Braće Branchetta 20, 51000 Rijeka, Croatia; valerija.majetic@medri.uniri.hr; 5 Faculty of Food Technology and Biotechnology, University of Zagreb, Pierottijeva 6, 10000 Zagreb, Croatia; miabrkljaca@gmail.com; 6 Department of Ecology, Agronomy and Aquaculture, University of Zadar, Mihovila Pavlinovića bb, 23000 Zadar, Croatia; simemarcelic@unizd.hr

**Keywords:** Croatia, cultivar, geographical location, nutrients, *Olea europaea* L., olive leaf, phenols, sampling time

## Abstract

The interactive effects of cultivar, collecting period, and geographical location on the content and composition of biophenols and macro and micronutrients in olive (*Olea europaea* L.) leaf were investigated. Leaves of six cultivars were collected at three periods in two locations in Croatia. The leaves of Istarska bjelica cultivar had the greatest biophenol (oleuropein) potential, especially those sampled in January and in March at the location of Pag. All the cultivars yielded leaves with the highest concentration of biophenols in March, which coincided with the pruning period. Except for high oleuropein concentration in Istarska bjelica, flavonoids were found to be most useful for differentiating olive leaves according to cultivar. Verbascoside turned out to be the most potent differentiator of collecting periods, while phosphorus and zinc turned out to be most useful for differentiating locations. Despite different agroecological conditions at the two locations, cultivar exhibited a significant effect on olive leaf nutrient composition, which was certainly causally related to that of the biophenols. The results obtained showed that it is possible to plan more well-timed and efficient exploitation of biophenols from olive leaf based on the knowledge about the interactive effects of the three studied factors.

## 1. Introduction

Olive (*Olea europaea* L.) and olive oil production generates high amounts of by-products and waste (olive pomace, leaves, stones, vegetable water) relative to the final product as well as in absolute global terms [1,2]. Olive and olive oil by-products are used mostly for the production of energy and animal feed. The so-called high-value use of olive biomass as a source of valuable bioactive compounds, that may be valorized in the functional food, cosmetic and pharmaceutical industries, is advancing rapidly in the recent years and is one of the priorities in the applied research in this field [1]. The compounds originating from olive leaf and its extracts are of special interest since they were shown to exhibit antioxidant [3,4,5,6], antimicrobial [7,8], hypoglycemic [9], lipid-lowering [5], and antihypertensive activity [10], and were attributed anti-HIV [11], anti-proliferative and apoptotic properties [12], protective effect against human leukemia [13] and anti-cancer inhibitive effect on tumor necrosis factor [14]. As well, olive leaf extracts have been studied as functional supplements to improve the quality and stability of meat products [15], vegetable oils [16], vegetable pâté [17], stracciatella cheese [18], and bread [19]. The olive leaf bioactive compounds most associated with such properties are biophenols belonging to the groups of simple phenolic alcohols, such as hydroxytyrosol and tyrosol, flavonoids, such as luteolin 7-*O*-glucoside, rutin, apigenin 7-*O*-glucoside and luteolin 4-*O*-glucoside, and secoiridoids, represented mainly by oleuropein, the most abundant phenolic component in olive leaf [20]. In order to provide in-depth information on their occurrence, numerous studies were conducted in the recent years with the aim to characterize olive leaf biophenolic profiles and to investigate the factors that affect them [2,21,22,23].

The recovery of biophenols from olive leaves might bring additional benefits to the sector by providing an additional source of income for producers and adding value to the supply chain [2], but the existing gaps in knowledge impede optimum targeted valorization of this potential. Although varietal origin has been shown to be a significant source of variability in olive leaf biophenol composition [2,21,22,23,24,25], cultivars grown in Croatia have been scarcely investigated from this aspect up to date [26]. Olive is an evergreen tree but its leaves are available in larger quantities during late winter and early spring as pruning residues or as by-products separated from fruits before processing in autumn, but only a few studies have taken this into consideration [22,24,26]. A recent study revealed a significant impact of geographical origin and showed that biophenols in olive leaf of particular cultivars are more sensible to pedoclimatic variations than others [27]. Therefore, it would be of practical importance to investigate the variation of olive leaf biophenols in different collecting periods at different olive growing locations, which was not done for Croatian cultivars yet, while the data at the global level are sporadical and insufficient to make more general conclusions.

In our previous report, we showed that temporal variation of olive leaf biophenolic composition may significantly depend on the cultivar [26]. The aim of this study was to further investigate the varietal diversity and to confirm the specificities of the biophenolic profiles in olive leaves from Croatian native and introduced cultivars, in order to better assess their particular potential as a source of these valuable compounds. Further, the aim was to investigate the influence of other important sources of variability, such as collection period and geographical location, to obtain a better insight into the behavior of biophenols in olive leaf through the season in different environments, which would allow planning their more well-timed and efficient exploitation. In addition to biophenols, the variability of the most important olive mineral macro- and micronutrients was monitored to provide valuable information about their status in Croatian olive cultivars particular agro-climatological conditions, which is crucial for optimal plant growth and significantly affects olive leaf biophenolic composition [28,29,30].

## 2. Results

### 2.1. Biophenols

The results of three-way analysis of variance (ANOVA) for biophenols in olive leaves, with cultivar, collecting period and location as factors, are presented in Table 1 and Table 2.

Among simple biophenols, Leccino leaves were the most abundant in hydroxytyrosol, whose concentration was the lowest in Istarska bjelica leaves (Table 1). High tyrosol concentration was characteristic for Drobnica leaves, followed by Leccino leaves, while the lowest concentration was found in Lastovka leaves. Vanillin was found in the highest concentration in Lastovka leaves, although not significantly different from that found in Leccino and Oblica leaves.

Leccino leaves contained higher concentration of 4-hydroxybenzoic acid than Istarska bjelica, Levantinka, and Oblica leaves. Lastovka leaves were the most abundant in caffeic, ferulic, and vanillic acid, although in some cases without statistical significance, while the concentrations found in Istarska bjelica leaves tended towards lower values. Lastovka leaves were distinguished from the others by the lowest concentration of verbascoside (Table 1).

Luteolin-7-*O*-glucoside was found in the highest concentration among flavonoids (Table 2). Leccino and Levantinka leaves contained the highest concentrations of apigenin and apigenin-7-*O*-glucoside, with the latter being more abundant in Leccino leaves. The highest concentration of luteolin and luteolin-7-*O*-glucoside were found in Levantinka leaves. Istarska bjelica leaves contained the lowest concentration of luteolin, although not significantly different than that found in Lastovka leaves, while the lowest concentration of its glucoside was noted in Drobnica, followed by Istarska bjelica and Lastovka leaves. Rutin concentration was the highest in Istarska bjelica, followed by Leccino and Drobnica leaves, while the lowest concentration found in Lastovka leaves was not significantly different from that found in Oblica leaves. Catechin was found in the highest concentration in Oblica leaves.

The highest oleuropein concentration was found in Istarska bjelica leaves. The total phenols concentration in Drobnica, Istarska bjelica, and Levantinka leaves was higher compared to that found in Lastovka and Oblica leaves (Table 2).

The concentrations of all the investigated simple biophenols exhibited a constant increase across the three studied collection periods from October 2017 (CP1) to January 2018 (CP2) and March 2018 (CP3), which was of higher intensity between CP1 and CP2 than between CP2 and CP3, except for tyrosol (Table 1). The changes in phenolic acids were more diverse. 4-Hydroxybenzoic, caffeic, and ferulic acid reached the lowest concentration at CP2, a constant decrease was noted for vanillic acid, while the increase in verbascoside concentration across the collection periods was rather sharp, especially from CP2 to CP3. The changes in the concentrations of flavonoid aglycons and glycosides were clearly distinct (Table 2). While apigenin and luteolin exhibited a constant decrease, the concentration of glycosides increased at a certain point. The increase in the concentration of apigenin-7-*O*-glucoside with respect to CP2 was noted at CP3, while the concentration of luteolin-7-*O*-glucoside and rutin increased at CP2 in relation to CP1. The increase in catechin concentration was rather constant. Oleuropein was characterized by a constant increase, while the concentrations of total phenols increased from CP2 to CP3.

The main effect of location was significant for the majority of biophenols, with higher concentration of hydroxytyrosol, vanillin, apigenin-7-*O*-glucoside, luteolin-7-*O*-glucoside, rutin, catechin, and oleuropein found in leaves from the orchard in Pag, and higher concentration of vanillic acid and apigenin in leaves from the orchard in Zadar (Table 1 and Table 2).

**Figure 1:**
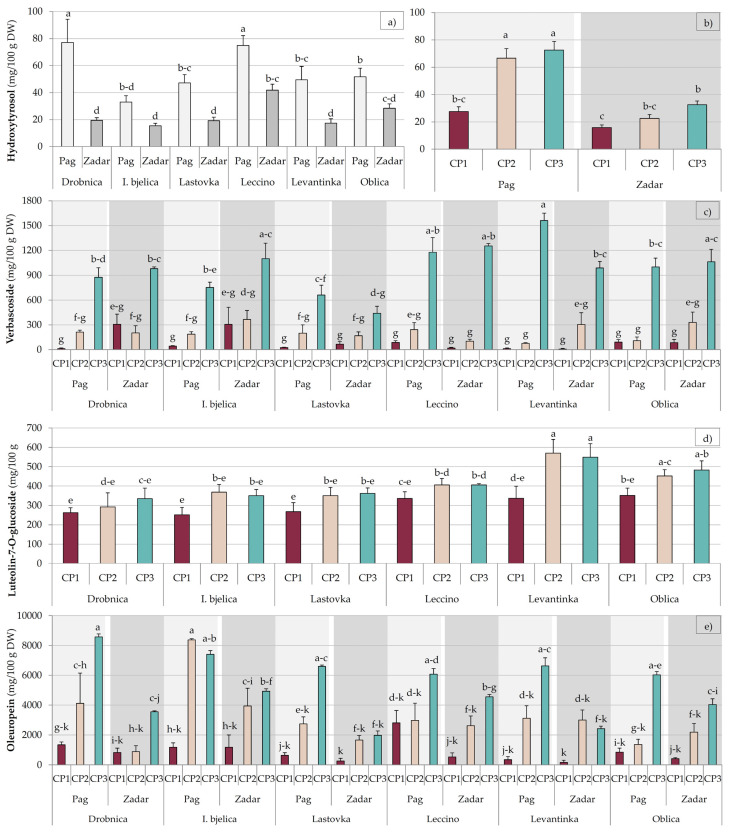
Multiple comparisons of the effects of cultivar, collection period, and location combinations (highest order interactions) on the concentrations of (**a**,**b**) hydroxytyrosol, (**c**) verbascoside, (**d**) luteolin-7-*O*-glucoside, and (**e**) oleuropein in leaves of Drobnica, Istarska bjelica (I. bjelica), Lastovka, Leccino, Levantinka, and Oblica olive cultivars collected at different periods (CP1—October 2017, CP2—January 2018, and CP3—March 2018) in two different locations (Pag and Zadar) in Croatia (burgundy, beige, and turquoise colors of histogram bars represent collecting periods CP1, CP2, and CP3, respectively, while lighter and darker gray colors (histogram bars and backgrounds) represent Pag and Zadar locations, respectively). Different superscript lowercase letters represent statistically significant differences between mean values at *p* < 0.05 obtained by a one-way ANOVA and Tukey’s test.

For many biophenols significant first and/or second order interactions were observed, suggesting the main effects and/or the interactions of lower order should be taken with caution (Table 1 and Table 2). Nevertheless, the main effects explained most of the changes that occurred. In most cases, multiple comparisons of means revealed similar patterns across the three factors for the majority of biophenols despite the significant interactions (Figure 1, Figures S1–S3).

The interaction of collecting period and location was noted for hydroxytyrosol, with an increase in its concentration from CP1 to CP2 in Pag which was not noted in Zadar. As well as this, the differences between the average concentrations at a particular location were differently expressed depending on the cultivar (Figure 1a,b). The main effect of collecting period was significant for tyrosol, however multiple comparisons revealed it was mainly due to the increase in its concentration in Levantinka leaves. A significant increase in tyrosol concentration at CP3 was observed only in Zadar (Figure S1). The concentration of vanillin increased from CP2 to CP3 in Pag, and from CP1 to CP2 in Zadar (Figure S1).

Multiple comparisons revealed that a decrease in 4-hydroxybenzoic acid concentration from CP1 to CP2 was most pronounced for Istarska bjelica leaves in Pag, for Lastovka and Leccino leaves in Zadar, and for Levantinka leaves at both locations (Figure S2). It was also revealed that the highest concentration of caffeic acid at CP3 was mostly owed to a large increase observed in Lastovka leaves at this collection period (Figure S2). It was clear that the highest concentration of vanillic acid at CP1 and the lowest at CP3 was mostly due to such changes observed in olive leaves in Zadar, especially for Lastovka, Levantinka, and Oblica cultivars (Figure S2). The lowest concentration of verbascoside in Lastovka leaves was mainly due to the lowest increase observed at CP3 for this in comparison to the other cultivars, especially in Zadar (Figure 1c).

The highest concentration of apigenin in Leccino and Levantinka leaves was mostly due to high initial concentration at CP1 in Zadar. A decrease in concentration noted for this compound across the collecting periods was more pronounced in Zadar for the majority of cultivars (Figure S3). Somewhat diverse behavior at the two locations was noted for luteolin: While in Pag its concentration dropped most significantly at CP3, in Zadar this occurred at CP2 (Figure S3). Multiple comparisons showed that the most significant increase in luteolin-7-*O*-glucoside concentration in Levantinka leaves occurred at CP2 and then it remained steady at CP3 in Zadar (Figure 1d), which was obviously the main reason for the highest concentration of this biophenol in leaves of this cultivar (Table 2). The highest concentration of rutin in Istarska bjelica leaves was mostly due to its rather high concentration found in Pag (Figure S3). A second-order interaction (cultivar × collecting period × location) was determined for catechin. Its concentration in Istarska bjelica leaves increased from CP1 to CP2 and stayed even to CP3 in Pag, while in Zadar an increase from CP2 to CP3 was observed. The concentration of catechin in Leccino leaves from Pag increased at CP3 in relation to CP1, which was not the case in Zadar (Figure S3).

For oleuropein, all the first and second order interactions were significant. Specific dynamics of the changes of its concentration were noted for Istarska bjelica leaves: A large increase was observed already at CP2, especially in Pag, with relatively steady concentration remaining at CP3. Similar was observed for Lastovka and Levantinka leaves in Zadar, although the concentrations in the leaves of these cultivars were lower. For the other cultivars a more gradual and constant increase was noted, expressed more at CP3 (Figure 1e). The pattern of the changes in total biophenol concentration observed at the two locations was different. While the concentration in Pag increased more or less gradually, for all the cultivars in Zadar the lowest leaf concentration was determined at CP2, although not always with statistical significance (Figure S3).

### 2.2. Mineral Nutrients

Considering the main effect, Drobnica leaves had the lowest concentration of P among the investigated cultivars (Table 3). The concentration of K was the highest in Drobnica and Oblica leaves, and the lowest in Levantinka leaves. Lastovka, Leccino, and Levantinka leaves were the most abundant in Ca, while Drobnica leaves contained the lowest concentration of this nutrient. Similar was observed for Mg. Lastovka leaves stood out with the highest Fe concentration, while Leccino and Oblica leaves had higher concentration of this nutrient than Levantinka leaves. The highest concentration of Zn was determined in Istarska bjelica and Oblica leaves. Leccino leaves were the most, and Drobnica and Lastovka leaves the least abundant in Mn. The concentration of Cu was higher in Leccino leaves than in Drobnica, Lastovka, and Oblica leaves. The highest concentration of Na was determined in Drobnica and Istarska bjelica leaves, although not significantly different from that found in Leccino leaves.

Several distinct patterns of the changes in the concentrations of the nutrients across the three collecting periods were noted. The concentration of P, Ca, and Fe were similar at CP1 and CP2 and then decreased at CP3 (Table 3). These fluctuations were relatively mild. Similar was observed for K, with the difference its concentration decreased steadily across the three collecting periods. Magnesium and Zn concentration increased from CP1 to CP2 and remained steady at CP3, while that of Cu exhibited a constant increase. The concentrations of Mn and B decreased from CP1 to CP2 and then increased from CP2 to CP3, with the highest concentration at CP1, while Na showed an opposite behavior, with a peak in concentration at CP2.

Phosphorus, K, B, and Na were more abundant in the leaves from Pag, while the concentrations of Ca, Mg, Fe, and Zn were higher in the leaves from Zadar location (Table 3).

**Figure 2:**
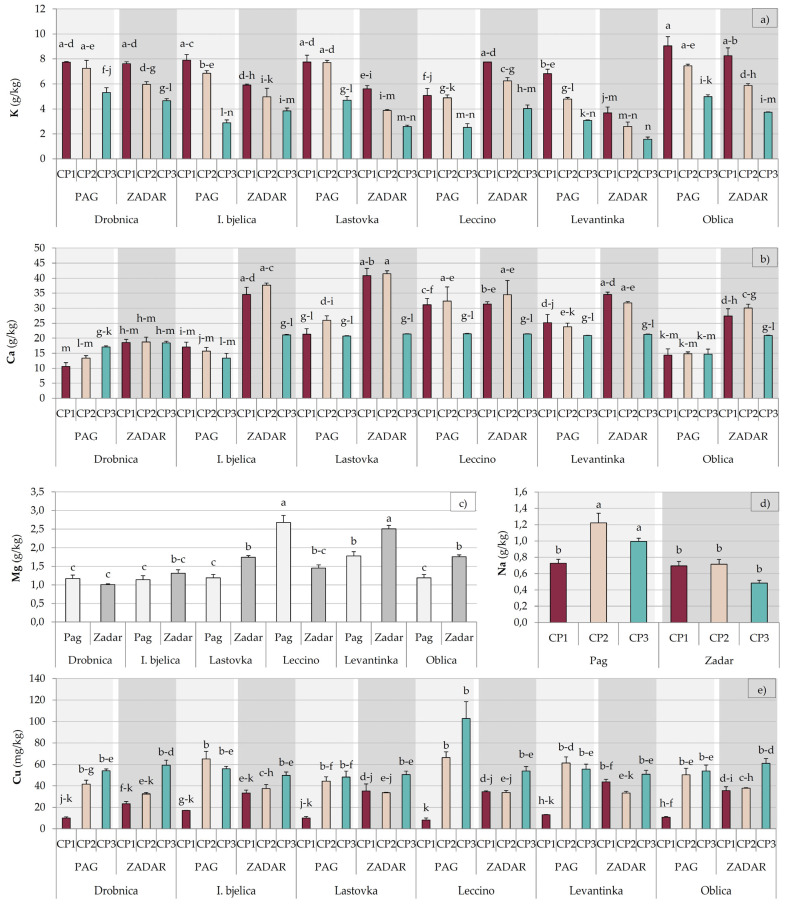
Multiple comparisons of the effects of cultivar, collection period, and location combinations (highest order interactions) on the concentrations of (**a**) potassium (K), (**b**) calcium (Ca), (**c**) magnesium (Mg), (**d**) sodium (Na), and (**e**) copper (Cu) concentrations in leaves of Drobnica, Istarska bjelica (I. bjelica), Lastovka, Leccino, Levantinka, and Oblica olive cultivars collected at different periods (CP1—October 2017, CP2—January 2018, and CP3—March 2018) in two different locations (Pag and Zadar) in Croatia (burgundy, beige, and turquoise colors of histogram bars represent collecting periods CP1, CP2, and CP3, respectively, while lighter and darker gray colors (histogram bars and backgrounds) represent Pag and Zadar locations, respectively). Different superscript lowercase letters represent statistically significant differences between mean values at *p* < 0.05 obtained by a one-way ANOVA and Tukey’s test.

Significant first and second order interactions were determined for all the nutrients (Table 3). A decrease in the concentration of P at CP3 was mainly due to the fluctuations which occurred in Zadar (Figure S4). Potassium level decreased during the time in leaves of all the investigated cultivars at both locations, although in some cases more significantly than in others (Figure 2a). The concentration of Ca was notably affected by the interactive effect of all the three studied factors. While for the majority of cultivar × collecting period × location combinations a drop in concentration was noted at CP3, Drobnica leaves, both in Pag and in Zadar, and Lastovka and Oblica leaves in Pag showed much less fluctuation (Figure 2b). A significant first order interaction of cultivar and location was determined for Mg. While location did not affect its concentration in Drobnica and Istarska bjelica leaves, the leaves of Lastovka, Levantinka, and Oblica had higher concentration in Zadar, while Leccino leaves contained more Mg in Pag (Figure 2c). The interactive effect of the three studied factor on the concentration of Fe was relatively strong, without specific patterns repeating (Figure S4). Higher concentration of Zn was found in Zadar, except in Leccino leaves (Figure S4). The increase in the concentration of Na at CP2 was mostly due to such fluctuation in the leaves from Pag (Figure 2d). The concentration of Cu was significantly affected by the interactive effect of all the three factors. A significant increase from CP1 to CP2 was observed in the leaves from Pag for all the cultivars, while in leaves from Zadar a milder increase was noted mostly at CP3 (Figure 2e). The interaction between cultivar, collecting period, and location was significant for B concentration (Table S4).

### 2.3. Multivariate Differentiation

**Figure 3:**
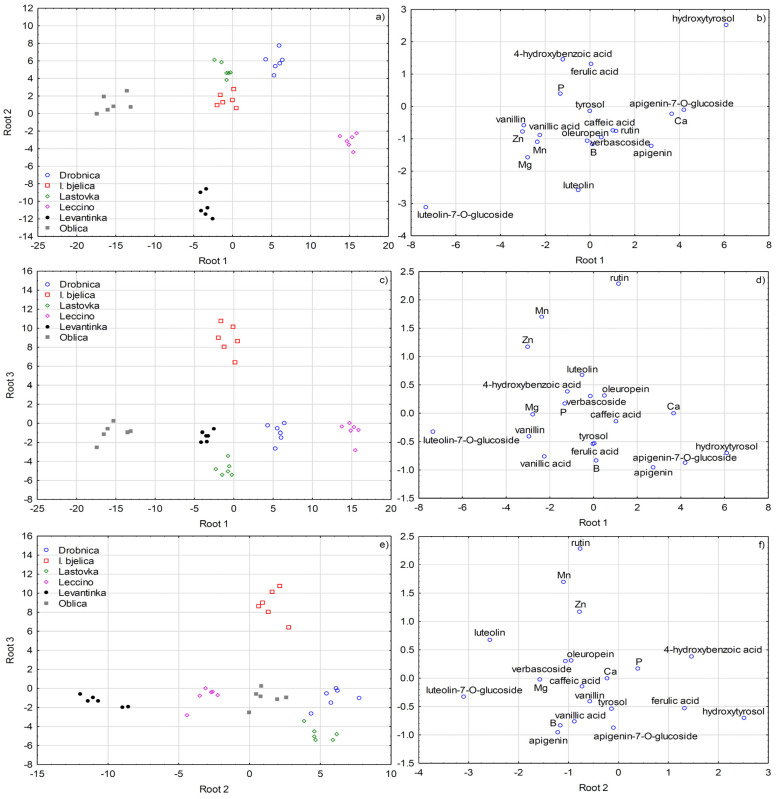
(**a**,**c**,**e**) Separation of olive leaves according to cultivar defined by the first three discriminant functions (roots) obtained by forward stepwise discriminant analysis (SLDA). (**b**,**d**,**f**) Standardized coefficients of biophenols and mineral nutrients selected by SLDA on the first three discriminant functions (roots).

**Figure 4:**
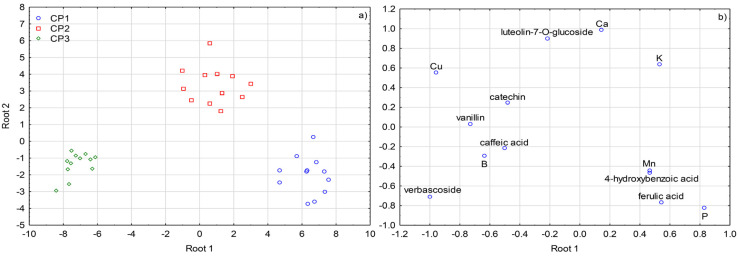
(**a**) Separation of olive leaves according to collecting period defined by the first two discriminant functions (roots) obtained by forward stepwise discriminant analysis (SLDA). (**b**) Standardized coefficients of biophenols and mineral nutrients selected by SLDA on the first two discriminant functions (roots). Collecting periods: CP1—October 2017, CP2—January 2018, and CP3—March 2018.

Stepwise linear discriminant analysis (SLDA) (Figure 3 and Figure 4) was applied on the dataset including 25 variables (concentrations of biophenols and nutrients) for the three factors separately. Each of the three SLDA differentiation models classified correctly all the olive leaves samples.

With cultivar as a grouping variable, a 100% correct classification was obtained after including five variables. Apigenin-7-*O*-glucoside entered model the first and correctly classified 41.67% of all the samples, while the inclusion of apigenin, tyrosol, rutin, and luteolin-7-*O*-glucoside achieved a 100% correct classification. The following variables improved the classification efficacy of the model, in a decreasing order according to their contribution: Zn, Mn, hydroxytyrosol, ferulic acid, Mg, Ca, vanillic acid, P, verbascoside, luteolin, oleuropein, caffeic acid, 4-hydroxybenzoic acid, and B. A clear visual differentiation of olive leaves according to cultivar defined by the first three discriminant functions (roots) was obtained (Figure 3). Most apparently, Drobnica and Leccino leaves were separated from those of the other cultivars along root 1, and were connected to low concentration of luteolin-7-*O*-glucoside in the former, and high hydroxytyrosol concentration in the letter, respectively, which basically corresponded to the ANOVA results (Table 1 and Table 2). Levantinka leaves were mostly differentiated by root 2 and were related to higher luteolin and luteolin-7-*O*-glucoside concentrations. Root 3 contributed mostly to the separation of Istarska bjelica leaves due to high concentrations of rutin, Zn, and Mn.

Verbascoside emerged as a most potent differentiator according to collecting period, since after its inclusion in the corresponding model 83.33% of all the samples were classified correctly. After including B, vanillin, 4-hydroxybenzoic acid, caffeic acid, Cu, P, catechin, and ferulic acid, the percentage of correctly classified samples increased to 100%, while luteolin-7-*O*-glucoside, Mn, K, and Ca further improved the classification efficacy of the model. Separation of olive leaf samples displayed in two dimensions of Cartesian plane was also very successful (Figure 4) and the connection between collecting periods and the concentrations of the variables very well reflected the results obtained by ANOVA (Table 1, Table 2 and Table 3). High concentrations of Mn, P, 4-hydroxybenzoic and ferulic acid were characteristic for CP1, CP2 was related to high concentrations of luteolin-7-*O*-glucoside, Ca and K, while the abundance in verbascoside, followed by vanillin, caffeic acid, catechin, and Cu, mostly contributed to the clear separation of CP3 samples.

When location was used as a grouping variable, the inclusion of only P and Zn as variables in the SLDA model was sufficient to correctly classify all the samples. Other variables included in the model were apigenin, tyrosol, catechin, K, Cu, Na, B, luteolin-7-*O*-glucoside, Mg, oleuropein, caffeic acid, and ferulic acid. The variables most related to the location in Pag were P, Na, and B, while the location in Zadar was mainly characterized by high concentration of Zn and apigenin, which was in accordance with the ANOVA results (Table 1, Table 2 and Table 3). The graphical representation of this separation was not possible since canonical analysis could not be performed because the number of levels for this factor was only two.

## 3. Discussion

The results of this study showed that the content and composition of biophenols in olive leaf significantly depend on cultivar, collecting period, and geographical location. Certain general characteristics of the composition of biophenols in olive leaf were found to be common for all the investigated cultivars. Oleuropein was found to be the most abundant phenol in leaves of all the investigated cultivars (Table 2), which corroborated the findings published for many other olive cultivars worldwide [2,21,22,24,25,26,31]. Luteolin-7-*O*-glucoside was predominant among flavonoids, which was in accordance with previous results for particular Spanish [21] and Portuguese cultivars [32], for particular cultivars grown in Morocco [2], as well as for the same Croatian cultivars as those investigated in this study but grown at another location [26]. High concentration of verbascoside (Table 1), a sugar ester of hydroxytyrosol and caffeic acid [21] developed by partial degradation of oleuropein [33], also coincided with the results of previous studies.

Significant differences between leaves of the investigated olive cultivars were determined for the concentration of each of the identified biophenols (Table 1 and Table 2, Figure 1). Many previous studies have shown that genotype is indeed one of the major factors of variability in olive leaf biophenol composition [2,21,22,23,24,25,26]. It was demonstrated recently that the activity of the two major enzymes responsible for phenol synthesis and oxidative degradation, phenylalanine ammonia-lyase (PAL) and polyphenol oxidase (PPO), respectively, is strongly cultivar dependent. Further, it was shown that PAL and PPO activities have a coordinated response, and that cultivars with high PAL efficiency exhibit low PPO efficiency and vice versa [24]. It is probable that the phenolic profiles of leaves of the investigated cultivars were, among other, strongly affected by the corresponding genetically pre-determined PAL and PPO loads, their potential activity, and actual efficiency. It is worth mentioning that the differences among cultivars observed in this study corresponded to a great extent to those obtained previously by comparing the leaves of the same cultivars grown in another location in Croatia [26], confirming cultivar as one of the strongest factors in determining olive leaf biophenolic profile. Istarska bjelica was confirmed as a cultivar with the highest olive leaf oleuropein potential (Table 2). Olives and olive oil of this cultivar are also known for their characteristic abundance in phenols, including oleuropein aglycones [34,35], so the results of this study corroborated the reports which found a positive correlation between the concentration of biophenols in fruits and leaves of the same olive cultivar [24,36].

Mineral nutrient availability is essential for optimal PAL activity and biophenol synthesis [29,37,38]. The concentrations of the majority of the investigated minerals were above the corresponding deficiency and adequate levels according to the literature (Table 3). The exceptions were K, whose concentration was below the deficiency limit of 4 g/kg at CP3 in most cases, which is often the case in Mediterranean drylands and calcareous soils [39], and B with the concentration at CP2 below 19 mg/kg as the lower limit of the interval of adequate concentrations in olive leaf [30].

It was shown previously that nutrient uptake, translocation, and effective usage ability by the plant are also cultivar related [40,41]. These properties can be significantly affected by agroecological factors, but also in a cultivar dependent manner [28]. The macro- or micronutrient requirements of cultivars may differ and different cultivars may differently respond to element deficiency or toxicity stresses [28,42,43,44]. Dimassi et al. [45] and Jordao et al. [46] reported that the nutrient composition of leaves, nutrient uptake and their utilization ability in different cultivars grown in conditions with the same pedo-climatic parameters significantly differed. In this study, such diversity was best seen in the changes of the concentrations of Ca, which was rather different in Drobnica and Oblica compared to the majority of other cultivars (Figure 2). The results obtained in this study mostly did not correspond to the previous findings on the nutrient composition of leaves of the same olive cultivars grown in another location in Croatia [26], suggesting the effect of cultivar significantly interacted with the effects of other environmental and agronomic factors.

Magnesium (Mg) and Mn cations are key nutrients that ensure correct PAL function and therefore the synthesis of biophenols in olive leaf [29]. However, in a previous study it was shown that foliar fertilization by Mg, Mn, and B resulted with a decrease in the concentration of flavonoids in olive leaf [47]. In contrast to these findings [47] and the results of our previous investigation where a negative correlation between luteolin and Mg concentrations was observed [26], in this study higher concentration of flavonoids, such as luteolin and apigenin and their glucosides, was found in Leccino and Levantinka leaves which were also the richest in Mg and Mn (Table 2 and Table 3). Particular interesting relationships were confirmed, including an antagonism between K and Mg (*r* = −0.54, *p* < 0.001), previously reported by Hartmann, Uriu and Lilleland [48], and a positive correlation between Mg and Ca concentrations (*r* = 0.48, *p* < 0.001), previously reported by Stateras and Moustakas [49]. Leaves of the cultivars more abundant in K, such as Drobnica and Oblica, had lower Ca and Mg concentrations (Table 3).

The investigated cultivars showed different variations of olive leaf biophenols across the three collecting periods (Table 1 and Table 2, Figure 1 and Figure S1–S3), which is in accordance with previous studies showing cultivar-dependent evolution of olive leaf biophenolic composition during the season [22,24,26]. A previous study reported that the changes in PAL and PPO activity in olive leaf during its development are also significantly pre-determined by genotype. Moreover, the same authors implied the possibility that different PAL and/or PPO isoforms could be predominant in leaves of different cultivars and significantly condition the final enzymatic efficiency [24]. In this study, the concentrations of the majority of major biophenols increased in early winter at CP2 and continued to rise until CP3 in early spring, at both locations (Table 1 and Table 2). Previous studies have shown that the concentrations of major biophenols in olive leaf, such as secoiridoids including oleuropein and their degradation products, increase as a defense response to low air temperatures in winter [22,24], which was probably the main cause of the increase observed in this study. Ortega-García et al. [24] and Talhaoui et al. [22] reported that the dynamics of such changes are cultivar dependent and are associated with the resistance and tolerance of a given cultivar to environmental conditions and its need for reaction against external stressors, which are related to their genetic heritage [22,24]. In our previous study, Istarska bjelica emerged as a cultivar with a specific response of olive leaf biophenolic composition during the time, especially oleuropein, presumably due to different tolerance and mechanisms of coping with cold stress with respect to other cultivars [26]. In this study, a similar behavior of Istarska bjelica leaves was noted, although it was significantly affected by location, with a much stronger increase at PC2 in Pag than in Zadar (Figure 1). Higher concentration of sodium at CP2 (Table 3), especially in Pag (Figure 2), suggests that higher availability of sea salt ions Na+ and Cl- brought by the wind, which is a rather frequent phenomenon in olive orchards located by the shore of the island of Pag in this period of year [50], possibly caused a plant response similar to mild salt stress, which is known to induce the biosynthesis of phenols, especially oleuropein, in olive leaf [51]. It was assumed that this was quite possible, especially knowing the low altitude of the orchard in Pag (5 m) and its rather short distance the sea (346 m). Lower concentration of Ca, as well as higher Na/K ratio found in the leaves from Pag could have also been the symptoms of such a response similar to salt stress, as reported previously [44,52]. It should be noted that the concentration of Na in leaves of all the cultivars was lower than 0.2% (2 g/kg) which is considered as the lower limit of the concentration range that can induce a toxic effect [30]. The symptoms of salt toxicity which include burns on leaf tip, leaf tissue necrosis, and falling of leaves were not detected, but they are known to occur in an advanced stage of leaf status deterioration [53]. The average concentration of Na at CP2 in Pag was 0.12% leaf DW on the average (Figure 2), but it reached 0.17% leaf DW in Istarska bjelica leaves collected at CP2 at the same location (data not shown), which is very near the proposed limit of 0.2% leaf DW. The thesis that the higher concentration of Na caused the increase in the concentration of biophenols in Istarska bjelica leaves was corroborated by a strong positive correlation determined between the concentration of this nutrient and oleuropein (*r* = 0.64, *p* < 0.001). Tolerance to salt stress was previously shown to be cultivar dependent [44] and Istarska bjelica was among the cultivars with the highest intake of Na and its translocation to leaves after growing in nutrient solution containing 33, 66, and 100 mM of NaCl for three months [52].

Concentration of K was found to constantly decrease across the three collecting periods (Table 3, Figure 2) which is in accordance with the fact that low winter temperatures reduce K availability and mobility [49] and that K is consumed during resistance to cold stress by lowering the freezing temperature [54]. Such a pattern for K was observed in our previous investigation [41]. It was suggested earlier that K is important for the regulation of plant water status [30], meaning its deficiency observed at CP3 possibly increased the response of the plant to possible water stress and induced the intensification of biophenol metabolism. Calcium exhibited a similar pattern at CP3 in March, which coincided to that observed by Fernández-Escobar, Moreno and Garcia-Creus [55] and Stateras and Moustakas [49].

The variations in P concentration corresponded to those observed previously at similar developmental stages of olive leaf, with relatively high concentration found in October, slightly lower in January, followed by a decrease in March which coincided with the development of flower buds and inflorescence [49]. The study in question [49] was conducted with the leaves of Kothreiki olive cultivar grown in Eastern Greece, an area characterized by the typical Mediterranean climate very similar to that in Pag and Zadar locations in this work. Similar was observed by Fernández-Escobar et al. [55] in Southern Córdoba province in Spain. A decrease observed by the end of winter at CP3 was possibly partly a consequence of weaker P uptake under lower air and soil temperatures, as indicated previously [56].

The concentration of Mg significantly increased from CP1 to CP2, and then showed a tendency towards decrease at CP3, which was observed previously by Stateras and Moustakas [49]. The lowest level of Mn was observed in CP2 coinciding with a period of low temperatures, which possibly reduced the absorption of Mn by the trees, as suggested previously [49]. The fluctuations in the concentration of B across the collecting periods observed in this study (Table 3) differed from its relatively steady concentration during the winter, as noted by others [49], but was in line with the similar behavior of B noted in our previous study [26].

Geographical location was previously confirmed as an important factor of variation in olive leaf biophenol composition [27,57,58,59,60]. In general, higher concentrations were found in leaves from northern parts and at higher altitudes [59,60]. The two locations investigated in this study were within a short radius of less than 60 km. The location of the orchard in Pag was at a more northern position, but had a lower altitude (5 m) compared to that in Zadar (95 m), so it is probable that other factors had a greater effect on the higher content of the majority of biophenols in the leaves sampled therefrom (Table 3). Average daily temperatures and rainfall measured across the collection periods from the beginning of October 2017 until the end of March 2018 on the two locations followed rather similar dynamics (Figure S4), suggesting the effects of climate was negligible. On the other hand, slightly different soil characteristics could have had a significant effect (Table S1).

Among biophenols, location exhibited the highest impact, in terms of *F*-value, on the concentration of luteolin-7-*O*-glucoside (Table 1 and Table 2), which was in agreement with the results from Taamalli et al. [27] who observed geographical origin having the strongest influence of flavones.

Higher concentration of P in olive leaves in Pag than in Zadar (Table 3) possibly contributed to the observed differences in the concentration of particular biophenols between selected locations. It was shown previously that concentration of P correlates negatively with the concentration of oleuropein and total biophenols in olive leaves, mainly because P availability increases N accumulation, which is inversely proportional to the level of biophenols due to competition between synthesis of biophenols and proteins in the same shikimic acid pathway [61,62]. However, in this study a significant positive correlation between the concentrations of P and hydroxythyrosol (*r* = 0.39, *p* < 0.001) and luteolin-7-*O*-glucoside (*r* = 0.44, *p* < 0.001) was determined, respectively. Tekaya et al. [62] observed a similar response of hydroxytyrosol concentration to foliar application of fertilizers containing P, in contrast to oleuropein and total biophenol whose concentration decreased. It is possible that higher P concentration slowed down oleuropein synthesis, which resulted in preserved concentration of its precursor, hydroxytyrosol, in the leaves from Pag (Table 1 and Table 3).

Generally lower concentration of biophenols in leaves from Zadar (Table 1 and Table 2) might have been partly related to the higher concentration of Ca in these leaves compared to leaves from Pag (Table 3). Tekaya et al. [62] observed a decrease in biophenols following an increase in Ca concentration in olive leaf after foliar fertilization. Penel et al. [63] proposed that Ca indirectly activates peroxydases, enzymes involved in oxidative degradation of biophenols, which results with a decrease in their concentration, as was the case in this work.

The already mentioned presumed pre-salt stress reaction to higher Na concentration found in the leaves from Pag (Table 3) was possibly also a reason for the stronger plant defense response against such conditions, which resulted in the increased biophenol concentration observed at this location compared to Zadar (Table 1 and Table 2).

Many significant interactive effects of the three investigated factors pointed to the great complexity of the interrelationships between biophenols and nutrients and their response to various sources of variability. Nevertheless, SLDA showed that the information given in the genotype remained significantly preserved (Figure 3), which confirmed cultivar is one of the key factors that determine the content of biophenols in olive leaf. On the other hand, clear distinctness between the collecting periods determined by uni- and multivariate statistical analysis (Table 1, Table 2 and Table 3, Figure 4) confirmed the existence of biosynthetic pathways during olive plant development common for all the investigated cultivars.

## 4. Materials and Methods

### 4.1. Olive Leaf Sampling

Experiment was conducted in 7 to 8 years old orchards planted on Calcocambisol soil at two different locations (Novalja, Island of Pag 44°32′53″ N and Poličnik near Zadar 14°52′58″ E) under fertilization practice common in the area [26]. Standard analytical methods for soil analysis were used [64] and soil chemical properties are reported in Table S1. The Köppen climate classification (Cfa) defines both experimental locations as Cfa [65], which was additionally confirmed by similar climatic conditions recorded during the study period (Figure S4). Each of the selected orchards contained five among the most important indigenous olive cultivars in Croatia (Drobnica, Istarska bjelica, Lastovka, Levantinka, and Oblica), as well as Italian Leccino, a common allochthonous cultivar in Croatian orchards [66]. Only well developed and similarly conditioned trees were included into trial. Agronomic traits of the selected cultivars were described previously [26], except Croatian autochthonous Lastovka, which is a self-incompatible cultivar, ripens late, has high and constant productivity with medium size fruits and medium oil yield [67].

Leaves from the central part of the olive shoots were collected evenly around the tree in three sampling periods, during olive harvest on 20 October 2017 (CP1), winter dormancy on 23 January 2018 (CP2), and pruning on 21 March 2018 (CP3), in triplicates. All the samples were carefully washed up, air dried at 30 °C in a dryer (Memmert GmbH + Co.KG, Büchenbach, Germany) up to constant mass, and milled using a Retsch ZM 200 mill (Retsch GmbH, Haan, Germany) to fine powder before analysis [38].

### 4.2. Chemicals

Chemicals used were the same as reported in our previous study together with the details about vendors [26], with the addition of pure chemical standards of vanillin and 4-hydroxybenzoic, caffeic, ferulic, and vanillic acids, which were procured from Extrasynthese (Genay, France, EU), and hydrochloric acid which was procured by Normapur, VRW International (Randor, PA, USA).

### 4.3. Analysis of Biophenols

High-performance liquid chromatographv (HPLC) with simultaneous UV/Vis detection at four different wavelengths using a Thermo Ultimate 3000 HPLC System (ThermoFischer Scientific, Waltman, MA, USA) was applied for identification and quantification of phenols, as described previously [26]. Prior to HPLC analysis, olive leaf phenols were extracted according to the procedure published in our previous work [68]. Briefly, 500 mg of air dried and finely ground leaves were subjected to extraction with 80 % methanol with the aid of an ultrasonic bath for 20 min. A volume of 14 mL of the extract was centrifuged and the liquid phase was filtered using a cellulose acetate syringe filter with 0.45 μm pores. HPLC analysis setup and conditions were described in our previous study [26]. 4-Hydroxybenzoic acid, luteolin-7-*O*-glucoside, oleuropein, and vanillic acid were detected and quantified at 250 nm, hydroxytyrosol, tyrosol, vanillin, apigenin-7-*O*-glucoside, and catechin at 280 nm, caffeic acid, ferulic acid, verbascoside, and apigenin at 305 nm, and luteolin and rutin at 370 nm. The phenols were identified based on the comparison of their retention times with those of pure standards, and quantified by external standard method using the corresponding calibration curves [26]. An example of an olive leaf extract HPLC chromatogram recorded at 280 nm, used for identification and quantification of hydroxytyrosol, tyrosol, vanillin, apigenin-7-*O*-glucoside, and catechin, is reported in Figure S5.

### 4.4. Analysis of Mineral Nutrients

Mineral nutrients were analyzed as described in our previous study [26], by inductively coupled plasma mass spectrometry (ICP-MS; boron (B), copper (Cu), manganese (Mn), and zinc (Zn)) using a NexION 300x system (PerkinElmer Instruments, Waltham, MA, USA), flame atomic absorption spectrometry (FAAS; calcium (Ca), magnesium (Mg), potassium (K), and iron (Fe)) using a PerkinElmer AAS800 system (PerkinElmer Instruments, Waltham, MA, USA) and acetylene-air as an oxidant, and UV/Vis spectrophotometry for the analysis of phosphorus (P) using a Carry UV/Vis 50 spectrophotometer (Varian Inc., Palo Alto, CA, USA) according to Miller [69]. Prior to analysis, 500-mg olive leaf samples were air dried, finely ground, and then ashed to dryness at 550 °C for 8 h. The obtained ash was dissolved in heated 0.6 M hydrochloric acid, filtered, and quantitatively diluted with deionized water. More details about the extraction procedure were reported previously [26,49].

### 4.5. Statistical Analysis

A completely random design (n = 3) was set at each of the two locations. Three-way analysis of variance (ANOVA) was conducted, with cultivar, collection period, and location as factors. For multiple comparisons of means, Tukey’s post-hoc test was performed at *p* ≤ 0.05. Data were further processed by forward stepwise linear discriminant analysis (SLDA), with an intention to extract the variables (phenols and mineral nutrients) most useful to differentiate olive leaf samples according to the three factors. Prior to SLDA, the data were normalized by mean-centering. Wilk’s lambda was used for the selection of variables with a criterion *F*-value to enter = 1. Statistica v. 13.2 software (StatSoft Inc., Tulsa, OK, USA) was used for all the calculations.

## 5. Conclusions

The results obtained proved that the content and composition of biophenols in olive leaf significantly depend on cultivar, collecting period, and geographical location. Particular cultivars were characterized by different patterns of the development of leaf biophenols during the time and responded differently to agroecological conditions at the two locations, meaning the studied factors significantly interacted, which pointed to the need to take this into account when estimating the availability of biophenols. In general, it was confirmed that all the investigated cultivars yield most valuable leaf contents in terms of biophenol concentration in early spring, which is of practical importance since it could coincide with the pruning period and could be valorized with minimum additional effort. From the practical point of view, in this specific case, the leaves of Istarska bjelica cultivar sampled in winter dormancy or in early spring at Pag seem to have the greatest biophenol (oleuropein) potential and are the most valuable from this aspect. Except for high oleuropein concentration in Istarska bjelica, flavonoids were found to be most useful for differentiation of olive cultivars. Verbascoside emerged as a most potent differentiator according to collecting period, while particular mineral nutrients, such as P and Zn, turned out to be most useful for differentiating locations. Despite somewhat different agroecological conditions at the two locations, cultivar exhibited a significant effect on olive leaf macro and micronutrient composition, which was certainly causally related to that of the biophenols. The results obtained confirmed the hypothesis that it is possible to plan more well-timed and efficient exploitation of biophenols from olive leaf based on the knowledge about the biophenolic potential of particular cultivars and its response to alterations introduced by collecting period and location. Further research should focus on investigating the influence of other factors known to affect the biophenols and mineral nutrients in olive leaves, particularly harvest year and water availability.

## Figures and Tables

**Table 1:** Concentrations of simple phenols and phenolic acids (mg/100 g DW) in leaves of six olive cultivars collected at three different periods (CP1—October 2017, CP2—January 2018, and CP3—March 2018) in two locations.

Factor	Simple Phenols (mg/100 g DW)				Phenolic Acids (mg/100 g DW)				
	Hydroxytyrosol	Tyrosol	Vanillin		4-Hydroxybenzoic acid	Caffeic Acid	Ferulic Acid	Vanillic Acid	Verbascoside
**Cultivar (Cv.)**									
Drobnica	48.3 ± 10.91 ^ab^	13.06 ± 1.34 ^a^	1.20 ± 0.24 ^bc^		1.16 ± 0.17 ^b^	1.78 ± 0.28 ^b^	1.79 ± 0.31 ^b^	2.02 ± 0.31 ^bc^	431.46 ± 91.96 ^a^
Istarska bjelica	24.19 ± 3.25 ^d^	8.35 ± 0.55 ^bc^	0.96 ± 0.15 ^bc^		1.57 ± 0.29 ^ab^	1.39 ± 0.25 ^b^	0.84 ± 0.18 ^c^	1.70 ± 0.31 ^c^	459.37 ± 97.23 ^a^
Lastovka	33.19 ± 4.69 ^cd^	4.66 ± 0.42 ^d^	1.83 ± 0.25 ^a^		1.42 ± 0.22 ^ab^	3.91 ± 0.88 ^a^	2.85 ± 0.45 ^a^	3.28 ± 0.63 ^a^	260.75 ± 59.94 ^b^
Leccino	58.38 ± 5.77 ^a^	10.44 ± 0.49 ^ab^	1.54 ± 0.18 ^ab^		1.90 ± 0.36 ^a^	1.99 ± 0.29 ^b^	1.88 ± 0.21 ^b^	1.38 ± 0.19 ^c^	480.47 ± 130.35 ^a^
Levantinka	33.48 ± 6.36 ^cd^	6.84 ± 1.03 ^cd^	0.83 ± 0.13 ^c^		1.35 ± 0.27 ^b^	1.82 ± 0.19 ^b^	2.12 ± 0.22 ^ab^	1.99 ± 0.42 ^bc^	492.01 ± 144.52 ^a^
Oblica	40.05 ± 4.47 ^bc^	8.52 ± 0.55 ^bc^	1.37 ± 0.19 ^abc^		1.33 ± 0.16 ^b^	2.10 ± 0.23 ^b^	2.26 ± 0.24 ^ab^	2.87 ± 0.43 ^ab^	446.76 ± 107.55 ^a^
*p*-value	***	***	***		**	***	***	***	***
*F*-value	14.75	16.98	6.33		3.69	7.29	9.12	10.4	5.28
**Collecting period (CP)**									
CP1	21.64 ± 2.20 ^c^	8.16 ± 0.55 ^b^	0.74 ± 0.05 ^c^		2.60 ± 0.18 ^a^	2.08 ± 0.12 ^ab^	2.66 ± 0.24 ^a^	3.32 ± 0.37 ^a^	89.05 ± 24.25 ^c^
CP2	44.57 ± 5.27 ^b^	7.66 ± 0.78 ^b^	1.37 ± 0.14 ^b^		1.02 ± 0.09 ^b^	1.71 ± 0.21 ^c^	1.28 ± 0.13 ^c^	2.5 ± 0.22 ^b^	208.47 ± 24.32 ^b^
CP3	52.59 ± 4.78 ^a^	10.11 ± 0.74 ^a^	1.75 ± 0.17 ^a^		0.75 ± 0.02 ^b^	2.70 ± 0.5 ^a^	1.93 ± 0.22 ^b^	0.8 ± 0.0 ^c^	987.89 ± 54.33 ^a^
*p*-value	***	**	***		***	*	***	***	***
*F*-value	51.11	6.77	24		112.8	4.62	19.88	65.84	347.94
**Location (L)**									
Pag	55.57 ± 4.28 ^a^	8.11 ± 0.62	1.42 ± 0.12 ^a^		1.39 ± 0.13	2.22 ± 0.22	1.84 ± 0.15	1.89 ± 0.17 ^b^	407.4 ± 65.06
Zadar	23.63 ± 1.71 ^b^	9.18 ± 0.54	1.16 ± 0.11 ^b^		1.51 ± 0.16	2.11 ± 0.30	2.07 ± 0.21	2.52 ± 0.30 ^a^	449.54 ± 59.7
*p*-value	***	n.s.	*		n.s.	n.s.	n.s.	**	n.s.
*F*-value	151.61	3.43	4.85		1.22	0.18	1.56	11.76	1.95
**Cv. × CP**	n.s.	*	n.s.		**	***	n.s.	***	***
*F*-value	1.35	2.15	1.88		2.90	7.41	1.59	5.52	6.19
**Cv. × L**	***	n.s.	*		**	n.s.	*	**	**
*F*-value	4.76	1.30	2.74		3.54	0.61	2.94	3.75	3.83
**CP × L**	***	*	*		n.s.	n.s.	n.s.	**	n.s.
*F*-value	15.44	4.72	4.89		1.19	0.96	0.52	6.74	1.61
**Cv. × CP × L**	n.s.	n.s.	n.s.		*	n.s.	**	**	**
*F*-value	1.91		1.49		2.5	0.9	2.59	3.11	2.65

Results are expressed as means ± standard errors. Different superscript lowercase letters in a column represent statistically significant differences between mean values for each main effect at *p* < 0.05 obtained by a two-way ANOVA and Tukey’s test. First (Cv. × CP, Cv. × L, CP × L) and second order interactions (Cv. × CP × L) are presented. Significance: ***—*p* < 0.001, **—*p* < 0.01, *—*p* < 0.05. DW—dry weight.

**Table 2:** Concentrations of flavonoids, secoiridoids, and total phenols (mg/100 g DW) in leaves of six olive cultivars collected at three different periods (CP1—October 2017, CP2—January 2018, and CP3—March 2018) in two locations.

Factor	Flavonoids (mg/100 g DW)						Secoiridoids (mg/100 g DW)	Total Phenols (mg/100 g DW)
	Apigenin	Apigenin-7-*O*- glucoside	Luteolin	Luteolin-7-*O*- glucoside	Rutin	Catechin	Oleuropein	
**Cultivar (Cv.)**								
Drobnica	4.92 ± 0.79 ^b^	29.87 ± 2.96 ^c^	30.48 ± 3.04 ^b^	296.78 ± 30.19 ^d^	55.70 ± 5.77 ^b^	25.87 ± 3.99 ^b^	3214.87 ± 722.32 ^b^	4431.7 ± 224.82 ^a^
Istarska bjelica	2.73 ± 0.71 ^b^	34.05 ± 1.89 ^c^	20.59 ± 2.79 ^c^	323.39 ± 23.55 ^cd^	107.60 ± 11.67 ^a^	27.15 ± 4.40 ^b^	4497.35 ± 705.29 ^a^	4385.06 ± 213.58 ^a^
Lastovka	4.64 ± 1.12 ^b^	31.14 ± 2.38 ^c^	28.49 ± 3.40 ^bc^	326.72 ± 23.98 ^cd^	19.82 ± 2.98 ^d^	25.51 ± 2.97 ^b^	2307.22 ± 515.42 ^b^	3714.59 ± 178.10 ^b^
Leccino	10.33 ± 2.25 ^a^	91.75 ± 5.67 ^a^	36.49 ± 3.03 ^b^	382.37 ± 17.13 ^bc^	55.88 ± 4.48 ^b^	22.76 ± 2.49 ^b^	3258.03 ± 476.23 ^b^	4030.79 ± 217.40 ^ab^
Levantinka	10.28 ± 1.91 ^a^	62.85 ± 5.09 ^b^	53.74 ± 3.00 ^a^	485.70 ± 44.67 ^a^	35.66 ± 3.96 ^c^	24.68 ± 3.83 ^b^	2612.49 ± 549.09 ^b^	4414.42 ± 165.97 ^a^
Oblica	2.74 ± 0.60 ^b^	27.79 ± 1.26 ^c^	31.68 ± 2.36 ^b^	428.80 ± 25.52 ^ab^	28.29 ± 3.31 ^cd^	34.39 ± 5.69 ^a^	2472.81 ± 493.50 ^b^	3836.30 ± 191.08 ^b^
*p*-value	***	***	***	***	***	***	***	***
*F*-value	22.09	155.49	30.88	23.12	77.75	8.75	11.58	8.6
**Collecting period (CP)**								
CP1	10.60 ± 1.43 ^a^	41.83 ± 3.88 ^b^	44.26 ± 2.37 ^a^	301.23 ± 17.44 ^b^	43.16 ± 5.95 ^b^	10.72 ± 1.08 ^c^	871.71 ± 149.51 ^c^	3721.92 ± 147.90 ^b^
CP2	5.34 ± 0.66 ^b^	43.28 ± 4.70 ^b^	33.01 ± 2.29 ^b^	406.60 ± 24.38 ^a^	55.69 ± 7.17 ^a^	28.60 ± 1.78 ^b^	3079.25 ± 376.50 ^b^	3909.95 ± 118.95 ^b^
CP3	1.89 ± 0.29 ^c^	53.63 ± 5.20 ^a^	23.47 ± 2.13 ^c^	414.06 ± 21.44 ^a^	52.63 ± 6.04 ^a^	40.86 ± 2.71 ^a^	5230.42 ± 332.04 ^a^	4774.57 ± 106.21 ^a^
*p*-value	***	***	***	***	**	***	***	***
*F*-value	69.27	19.32	53.68	35	6.68	248.14	169.68	53.62
**Location (L)**								
Pag	4.49 ± 0.59 ^b^	52.90 ± 4.24 ^a^	32.81 ± 2.29	455.41 ± 17.20 ^a^	63.49 ± 6.22 ^a^	33.38 ± 2.38 ^a^	3950.63 ± 398.44 ^a^	4158.72 ± 142.94
Zadar	7.39 ± 1.09 ^a^	39.58 ± 3.10 ^b^	34.34 ± 2.05	292.51 ± 12.33 ^b^	37.49 ± 3.19 ^b^	20.08 ± 1.89 ^b^	2170.29 ± 227.20 ^b^	4112.24 ± 91.12
*p*-value	***	***	n.s.	***	***	***	***	n.s.
*F*-value	22.65	62.08	0.87	174.94	79.27	143.2	84.92	0.28
**Cv. × CP**	***	***	n.s.	*	n.s.	***	***	*
*F*-value	4.96	7.87	1.22	2.04	1.25	11.50	4.21	2.19
**Cv. × L**	n.s.	***	n.s.	***	***	**	*	*
*F*-value	1.48	6.09	1.36	5.48	21.81	3.46	3.08	2.76
**CP × L**	***	***	*	n.s.	n.s.	**	***	***
*F*-value	16.14	7.99	4.50	0.12	1.57	7.23	16.98	52.73
**Cv. × CP × L**	**	n.s.	n.s.	n.s.	n.s.	***	***	*
*F*-value	2.8	1.02	1.22	0.72	0.98	3.55	3.68	2.53

Results are expressed as means ± standard errors. Different superscript lowercase letters in a column represent statistically significant differences between mean values for each main effect at *p* < 0.05 obtained by a two-way ANOVA and Tukey’s test. First (Cv. × CP, Cv. × L, CP × L) and second order interactions (Cv. × CP × L) are presented. Significance: ***—*p* < 0.001, **—*p* < 0.01, *—*p* < 0.05. DW—dry weight.

**Table 3:** Concentrations of macro (g/kg) and micronutrients (mg/kg) in leaves of six olive cultivars collected at three different periods (CP1—October 2017, CP2—January 2018, and CP3—March 2018) in two locations.

Factor	Macronutrients (g/kg)					Micronutrients (mg/kg)				
	Phosphorous	Potassium	Calcium	Magnesium	Sodium	Iron	Zinc	Manganese	Copper	Boron
**Cultivar (Cv.)**										
Drobnica	1.20 ± 0.04 ^b^	6.42 ± 0.31 ^a^	16.14 ± 0.83 ^c^	1.09 ± 0.05 ^c^	0.98 ± 0.08 ^a^	77.93 ± 2.53 ^bc^	24.04 ± 1.45 ^b^	48.86 ± 2.17 ^c^	36.73 ± 4.24 ^b^	19.79 ± 0.88
Istarska bjelica	1.34 ± 0.04 ^a^	5.39 ± 0.43 ^b^	23.25 ± 2.34 ^b^	1.23 ± 0.07 ^bc^	0.95 ± 0.10 ^a^	77.10 ± 2.14 ^bc^	29.87 ± 1.96 ^a^	56.99 ± 4.10 ^b^	43.02 ± 4.07 ^ab^	19.72 ± 0.97
Lastovka	1.33 ± 0.05 ^a^	5.37 ± 0.47 ^b^	28.62 ± 2.24 ^a^	1.47 ± 0.08 ^b^	0.71 ± 0.09 ^bc^	91.68 ± 3.79 ^a^	24.79 ± 1.11 ^b^	44.46 ± 1.72 ^c^	36.93 ± 3.58 ^b^	19.68 ± 1.10
Leccino	1.33 ± 0.04 ^a^	5.08 ± 0.41 ^b^	28.71 ± 1.61 ^a^	2.07 ± 0.18 ^a^	0.89 ± 0.07 ^ab^	78.67 ± 2.99 ^b^	25.78 ± 0.87 ^b^	67.87 ± 2.74 ^a^	49.82 ± 7.63 ^a^	18.26 ± 1.11
Levantinka	1.30 ± 0.05 ^a^	3.76 ± 0.42 ^c^	26.23 ± 1.33 ^a^	2.14 ± 0.11 ^a^	0.64 ± 0.07 ^c^	68.70 ± 2.63 ^c^	25.22 ± 1.35 ^b^	56.92 ± 2.09 ^b^	42.89 ± 4.09 ^ab^	19.61 ± 1.09
Oblica	1.37 ± 0.04 ^a^	6.56 ± 0.47 ^a^	20.38 ± 1.63 ^b^	1.47 ± 0.09 ^b^	0.68 ± 0.04 ^bc^	80.69 ± 3.99 ^b^	31.38 ± 2.35 ^a^	51.34 ± 2.14 ^bc^	41.52 ± 4.22 ^b^	19.22 ± 0.76
*p*-value	***	***	***	***	***	***	***	***	***	n.s.
*F*-value	9.08	54.30	50.77	49.02	8.6	10.54	19.17	20.04	6.98	2.1
**Collecting period (CP)**										
CP1	1.36 ± 0.02 ^a^	6.93 ± 0.27 ^a^	25.59 ± 1.57 ^a^	1.41 ± 0.10 ^b^	0.71 ± 0.04 ^b^	79.86 ± 2.32 ^a^	25.40 ± 1.43 ^b^	61.86 ± 2.25 ^a^	22.80 ± 2.17 ^c^	22.92 ± 0.72 ^a^
CP2	1.33 ± 0.04 ^a^	5.7 ± 0.26 ^b^	26.68 ± 1.61 ^a^	1.73 ± 0.09 ^a^	0.97 ± 0.08 ^a^	83.70 ± 2.45 ^a^	27.19 ± 1.21 ^a^	46.20 ± 1.53 ^c^	44.75 ± 2.30 ^b^	15.77 ± 0.24 ^c^
CP3	1.25 ± 0.03 ^b^	3.66 ± 0.19 ^c^	19.39 ± 0.49 ^b^	1.59 ± 0.10 ^a^	0.74 ± 0.05 ^b^	73.83 ± 2.23 ^b^	27.96 ± 0.86 ^a^	55.17 ± 1.94 ^b^	57.91 ± 2.77 ^a^	19.45 ± 0.39 ^b^
*p*-value	***	***	***	***	***	***	**	***	***	***
*F*-value	17.51	286.44	63.31	13.50	15.48	9.50	7.25	37.10	188.57	156.23
**Location (L)**										
Pag	1.46 ± 0.02 ^a^	5.93 ± 0.27 ^a^	19.66 ± 0.90 ^b^	1.52 ± 0.09 ^b^	0.98 ± 0.05 ^a^	73.79 ± 1.63 ^b^	21.9 ± 0.57 ^b^	54.95 ± 1.93	42.63 ± 3.64	19.86 ± 0.63 ^a^
Zadar	1.17 ± 0.02 ^b^	4.93 ± 0.26 ^b^	28.11 ± 1.12 ^a^	1.63 ± 0.07 ^a^	0.63 ± 0.03 ^b^	84.46 ± 2.02 ^a^	31.8 ± 0.82 ^a^	53.87 ± 1.65	41.01 ± 1.56	18.90 ± 0.49 ^b^
*p*-value	***	***	***	*	***	***	***	n.s.	n.s.	**
*F*-value	317.5	79.27	219.38	4.55	70.94	32.78	308.42	0.53	1.19	8.38
**Cv. × CP**	**	*	***	n.s.	n.s.	n.s.	*	n.s.	***	***
*F*-value	2.78	2.19	6.68	1.11	1.90	1.83	1.99	1.49	4.38	6.61
**Cv. × L**	n.s.	***	***	***	n.s.	*	***	***	***	***
*F*-value	0.82	33.55	15.61	34.58	1.72	3.28	16.96	5.20	6.58	12.20
**CP × L**	***	**	***	n.s.	***	*	***	*	***	***
*F*-value	18.38	7.49	25.21	2.94	14.56	3.29	19.67	3.17	72.67	10.03
**Cv. × CP × L**	n.s.	**	*	n.s.	n.s.	*	n.s.	n.s.	***	***
*F*-value	0.98	3.24	2.09	0.34	1.79	2.13	0.50	1.49	4.13	7.54

Results are expressed as means ± standard errors. Different superscript lowercase letters in a column represent statistically significant differences between mean values for each main effect at *p* < 0.05 obtained by a two-way ANOVA and Tukey’s test. First (Cv. × CP, Cv. × L, CP × L) and second order interactions (Cv. × CP × L) are presented. Significance: ***—*p* < 0.001, **—*p* < 0.01, *—*p* < 0.05. DW—dry weight.

## Supplementary Materials

The following are available online at https://www.mdpi.com/2223-7747/9/12/1667/s1, Figure S1. Multiple comparisons of the effects of cultivar, collection period, and location combinations (highest order interactions) on the concentrations of (**a**,**b**) tyrosol, (**c**) caffeic acid, (**d**) vanillin, (**e**) 4-hydroxybenzoic acid, (**f**) vanillic acid, and (**g**) ferulic acid in leaves of Drobnica, Istarska bjelica, Lastovka, Leccino, Levantinka, and Oblica olive cultivars collected at different periods (CP1—October 2017, CP2—January 2018, and CP3—March 2018) in two different locations (Pag and Zadar) in Croatia. Different superscript lowercase letters represent statistically significant differences between mean values at *p* < 0.05 obtained by a one-way ANOVA and Tukey’s test; Figure S2. Multiple comparisons of the effects of cultivar, collection period, and location combinations (highest order interactions) on the concentrations of (**a**–**c**) apigenin-7-O-glucoside, (**d**) rutin, (**e**) luteolin, (**f**) catechin, (**g**) apigenin, and (**h**) total phenols in leaves of Drobnica, Istarska bjelica, Lastovka, Leccino, Levantinka, and Oblica olive cultivars collected at different periods (CP1—October 2017, CP2—January 2018, and CP3—March 2018) in two different locations (Pag and Zadar) in Croatia. Different superscript lowercase letters represent statistically significant differences between mean values at *p* < 0.05 obtained by a one-way ANOVA and Tukey’s test; Figure S3. Multiple comparisons of the effects of cultivar, collection period, and location combinations (highest order interactions) on the concentrations of (**a**,**b**) phosphorous (P), (**c**) iron (Fe), (**d**–**f**) zinc (Zn), (**g**,**h**) manganese (Mn), and (**i**) boron (B) concentrations in leaves of Drobnica, Istarska bjelica, Lastovka, Leccino, Levantinka, and Oblica olive cultivars collected at different periods (CP1—October 2017, CP2—January 2018, and CP3—March 2018) in two different locations (Pag and Zadar) in Croatia. Different superscript lowercase letters represent statistically significant differences between mean values at *p* < 0.05 obtained by a one-way ANOVA and Tukey’s test; Figure S4. Average daily rainfall (mm) and temperatures (°C) measured from the beginning of October 2017 until the end of March 2018 on the experiment locations (**a**) Pag and (**b**) Zadar with indicated olive leaf collecting periods (CP1–CP3). Figure S5. Example of an olive leaf extract HPLC chromatogram recorded at 280 nm used for identification and quantification of hydroxytyrosol, tyrosol, vanillin, apigenin-7-*O*-glucoside, and catechin. Table S1. Chemical properties of the Calcocambisol soil at two locations (Novalja, Island of Pag 44°32′53″ N and Poličnik near Zadar 14°52′58″ E) used in the study;

## References

[1] Berbel J., Posadillo A. (2018). Review and analysis of alternatives for the valorisation of agro-industrial olive oil by-products. Sustainability.

[2] Olmo-Garcia L., Bajoub A., Benlamaalam S., Hurtado-Fernandez E., Bagur-Gonzalez M.G., Chigr M., Mbarki M., Fernandez-Gutierrez A., Carrasco-Pancorbo A. (2018). Establishing the phenolic composition of *Olea europaea* L. leaves from cultivars grown in Morocco as a crucial step towards their subsequent exploitation. Molecules.

[3] Bulotta S., Corradino R., Celano M., D’Agostino M., Maiuolo J., Oliverio M., Procopio A., Iannone M., Rotiroti D., Russo D. (2011). Antiproliferative and antioxidant effects on breast cancer cells of oleuropein and its semisynthetic peracetylated derivatives. Food Chem..

[4] Jemai H., El Feki A., Sayadi S. (2009). Antidiabetic and antioxidant effects of hydroxytyrosol and oleuropein from olive leaves in alloxan-diabetic rats. J. Agric. Food Chem..

[5] Jemai H., Fki I., Bouaziz M., Bouallagui Z., El Feki A., Isoda H., Sayadi S. (2008). Lipid-lowering and antioxidant effects of hydroxytyrosol and its triacetylated derivative recovered from olive tree leaves in cholesterol-fed rats. J. Agric. Food Chem..

[6] Lee O.H., Lee B.Y. (2010). Antioxidant and antimicrobial activities of individual and combined phenolics in *Olea europaea* leaf extract. Bioresour. Technol..

[7] Sudjana A.N., D’Orazio C., Ryan V., Rasool N., Ng J., Islam N., Riley T.V., Hammer K.A. (2009). Antimicrobial activity of commercial *Olea europaea* (olive) leaf extract. Int. J. Antimicrob. Agents.

[8] Korukluoglu M., Sahan Y., Yigit A. (2008). Antifungal properties of olive leaf extracts and their phenolic compounds. J. Food Saf..

[9] Somova L.I., Shode F.O., Mipando M. (2004). Cardiotonic and antidysrhythmic effects of oleanolic and ursolic acids, methyl maslinate and uvaol. Phytomedicine.

[10] Susalit E., Agus N., Effendi I., Tjandrawinata R.R., Nofiarny D., Perrinjaquet-Moccetti T., Verbruggen M. (2011). Olive (*Olea europaea*) leaf extract effective in patients with stage-1 hypertension: Comparison with Captopril. Phytomedicine.

[11] Lee-Huang S., Zhang L., Huang P.L., Chang Y.T. (2003). Anti-HIV activity of olive leaf extract (OLE) and modulation of host cell gene expression by HIV-1 infection and OLE treatment. Biochem. Biophys. Res. Commun..

[12] Han J., Talorete T.P.N., Yamada P., Isoda H. (2009). Anti-proliferative and apoptotic effects of oleuropein and hydroxytyrosol on human breast cancer MCF-7 cells. Cytotechnology.

[13] Abaza L., Talorete T.P.N., Yamada P., Kurita Y., Zarrouk M., Isoda H. (2007). Induction of growth inhibition and differentiation of human leukemia HL-60 cells by a Tunisian gerboui olive leaf extract. Biosci. Biotechnol. Biochem..

[14] Nishibe S., Han Y., Noguchi Y., Ueda H., Yamazaki M., Mizutani K., Kambara T., Kishida N. (2001). The inhibitory effects of the compounds from olive leaf on tumor necrosis factor production and on β-hexosaminidase release. J. Nat. Med..

[15] Aouidi F., Okba A., Hamdi M. (2017). Valorization of functional properties of extract and powder of olive leaves in raw and cooked minced beef meat. J. Sci. Food Agric..

[16] de Medina V.S., Priego-Capote F., de Castro M.D.L. (2012). Characterization of refined edible oils enriched with phenolic extracts from olive leaves and pomace. J. Agric. Food Chem..

[17] Difonzo G., Squeo G., Calasso M., Pasqualone A., Caponio F. (2019). Physico-chemical, microbiological and sensory evaluation of ready-to-use vegetable pâté added with olive leaf extract. Foods.

[18] Natrella G., Difonzo G., Calasso M., Costantino G., Caponio F., Faccia M. (2020). Evolution of VOC and sensory characteristics of stracciatella cheese as affected by different preservatives. Foods.

[19] Baiano A., Viggiani I., Terracone C., Romaniello R., Del Nobile M.A. (2015). Physical and sensory properties of bread enriched with phenolic aqueous extracts from vegetable wastes. Czech J. Food Sci..

[20] Sahin S., Bilgin M. (2018). Olive tree (*Olea europaea* L.) leaf as a waste by-product of table olive and olive oil industry: A review. J. Sci. Food Agric..

[21] Talhaoui N., Gomez-Caravaca A.M., Leon L., De la Rosa R., Segura-Carretero A., Fernandez-Gutierrez A. (2014). Determination of phenolic compounds of ’Sikitita ’ olive leaves by HPLC-DAD-TOF-MS. Comparison with its parents ’Arbequina’ and ’Picual’ olive leaves. Lwt Food Sci. Technol..

[22] Talhaoui N., Gomez-Caravaca A.M., Roldan C., Leon L., De la Rosa R., Fernandez-Gutierrez A., Segura-Carretero A. (2015). Chemometric analysis for the evaluation of phenolic patterns in olive leaves from six cultivars at different growth stages. J. Agric. Food Chem..

[23] Romero C., Medina E., Mateo M.A., Brenes M. (2017). Quantification of bioactive compounds in Picual and Arbequina olive leaves and fruit. J. Sci. Food Agric..

[24] Ortega-Garcia F., Peragon J. (2010). Phenol metabolism in the leaves of the olive tree (*Olea europaea* L.) cv. Picual, Verdial, Arbequina, and Frantoio during ripening. J. Agric. Food Chem..

[25] Ranalli A., Contento S., Lucera L., Di Febo M., Marchegiani D., Di Fonzo V. (2006). Factors affecting the contents of iridoid oleuropein in olive leaves (*Olea europaea* L.). J. Agric. Food Chem..

[26] Pasković I., Lukić I., Žurga P., Germek V.M., Brkljača M., Koprivnjak O., Major N., Grozić K., Franić M., Ban D. (2020). Temporal variation of phenolic and mineral composition in olive leaves is cultivar dependent. Plants.

[27] Taamalli A., Roman D.A., Caravaca A.M.G., Zarrouk M., Carretero A.S. (2018). Geographical characterization of Tunisian olive tree leaves (cv. Chemlali) using HPLC-ESI-TOF and IT/MS fingerprinting with hierarchical cluster analysis. J. Anal. Methods Chem..

[28] Toplu C., Uygur V., Yildiz E. (2009). Leaf mineral composition of olive varieties and their relation to yield and adaptation ability. J. Plant Nutr..

[29] Treutter D. (2010). Managing phenol contents in crop plants by phytochemical farming and breeding-visions and constraints. Int. J. Mol. Sci..

[30] Fernández-Escobar R. (2019). Olive nutritional status and tolerance to biotic and abiotic stresses. Front. Plant Sci..

[31] Fu S.P., Arráez-Roman D., Segura-Carretero A., Menéndez J.A., Menéndez-Gutierrez M.P., Micol V., Fernandez-Gutierrez A. (2010). Qualitative screening of phenolic compounds in olive leaf extracts by hyphenated liquid chromatography and preliminary evaluation of cytotoxic activity against human breast cancer cells. Anal. Bioanal. Chem..

[32] Meirinhos J., Silva B.M., Valentao P., Seabra R.M., Pereira J.A., Dias A., Andrade P.B., Ferreres F. (2005). Analysis and quantification of flavonoidic compounds from Portuguese olive (*Olea europaea* L.) leaf cultivars. Nat. Prod. Res..

[33] Artajo L.S., Romero M.P., Suarez M., Motilva M.J. (2007). Partition of phenolic compounds during the virgin olive oil industrial extraction process. Eur. Food Res. Technol..

[34] Lukić I., Horvat I., Godena S., Krapac M., Lukić M., Vrhovsek U., Brkić Bubola K. (2018). Towards understanding the varietal typicity of virgin olive oil by correlating sensory and compositional analysis data: A case study. Food Res. Int..

[35] Lukić I., Lukić M., Žanetić M., Krapac M., Godena S., Brkić Bubola K. (2019). Inter-varietal diversity of typical volatile and phenolic profiles of Croatian extra virgin olive oils as revealed by GC-IT-MS and UPLC-DAD analysis. Foods.

[36] Ortega-Garcia F., Blanco S., Peinado M.A., Peragon J. (2009). Phenylalanine ammonia-lyase and phenolic compounds in leaves and fruits of *Olea europaea* L. cv. Picual during ripening. J. Sci. Food Agric..

[37] Pasković I., Herak Ćustić M., Pecina M., Bronić J., Ban D., Radić T., Poščić F., Špika M.J., Soldo B., Palčić I. (2019). Manganese soil and foliar fertilization of olive plantlets: The effect on leaf mineral and phenolic content and root mycorrhizal colonization. J. Sci. Food Agric..

[38] Pasković I., Soldo B., Talhaoui N., Palčić I., Brkljača M., Koprivnjak O., Germek V.M., Ban D., Klanjac J., Franić M. (2019). Boron foliar application enhances oleuropein level and modulates volatile compound composition in olive leaves. Sci. Hortic..

[39] Restrepo-Diaz H., Benlloch M., Navarro C., Fernández-Escobar R. (2008). Potassium fertilization of rainfed olive orchards. Sci. Hortic..

[40] Chatzistathis T., Therios I., Alifragis D. (2009). Differential uptake, distribution within tissues, and use efficiency of manganese, iron, and zinc by olive cultivars kothreiki and koroneiki. Hortscience.

[41] Pasković I., Perica S., Pecina M., Hančević K., Polić Pasković M., Herak Ćustić M. (2013). Leaf mineral concentration of five olive cultivars grown on calcareous soil. J. Cent. Eur. Agric..

[42] Therios I.N., Misopolinos N.D. (1988). Genotypic response to sodium chloride salinity of four major olive cultivars (*Olea europaea* L.). Plant Soil.

[43] Marschner H. (2012). Marschner’s Mineral Nutrition of Higher Plants.

[44] Loupassaki M.H., Chartzoulakis K.S., Digalaki N.B., Androulakis I. (2002). Effects of salt stress on concentration of nitrogen, phosphorus, potassium, calcium, magnesium, and sodium in leaves, shoots, and roots of six olive cultivars. J. Plant Nutr..

[45] Dimassi K., Therios I., Passalis A. (1999). Genotypic effect on leaf mineral levels of 17 olive cultivars grown in Greece. Acta Hortic..

[46] Jordao P.V., Marcelo M.E., Centeno M.S.L. (1999). Effect of cultivar on leaf-mineral composition of olive tree. Acta Hortic..

[47] Ben Abdeljelil Z., Tekaya M., Elmsellem H., Mechri B., Hammami M. (2017). Impact of season and foliar fertilisers on phenolics of leaves of Chemlali olive cultivar. Moroc. J. Chem..

[48] Hartmann H., Uriu K., Lilleland O., Childers N. (1966). Olive nutrition. Fruit Nutrition.

[49] Stateras D.C., Moustakas N.K. (2018). Seasonal changes of macro- and micro-nutrients concentration in olive leaves. J. Plant Nutr..

[50] Tadejević V. (1987). Some experiences with the protection of field crops from sneet along Adriatic coast. Agron. Glas..

[51] Petridis A., Therios I., Samouris G., Tananaki C. (2012). Salinity-induced changes in phenolic compounds in leaves and roots of four olive cultivars (*Olea europaea* L.) and their relationship to antioxidant activity. Environ. Exp. Bot..

[52] Perica S., Goreta S., Vuletin Selak G. (2008). Growth, biomass allocation and leaf ion concentration of seven olive (*Olea europaea* L.) cultivars under increased salinity. Sci. Hortic..

[53] Tattini M., Bertoni P., Caselli S. (1992). Genotypic responses of olive plants to sodium-chloride. J. Plant Nutr..

[54] Wang M., Zheng Q.S., Shen Q.R., Guo S.W. (2013). The critical role of potassium in plant stress response. Int. J. Mol. Sci..

[55] Fernández-Escobar R., Moreno R., Garcia-Creus M. (1999). Seasonal changes of mineral nutrients in olive leaves during the alternate-bearing cycle. Sci. Hortic..

[56] Yevdokimov I., Larionova A., Blagodatskaya E. (2016). Microbial immobilisation of phosphorus in soils exposed to drying-rewetting and freeze-thawing cycles. Biol. Fertil. Soils.

[57] Japon-Lujan R., Ruiz-Jimenez J., De Castro M.D.L. (2006). Discrimination and classification of olive tree varieties and cultivation zones by biophenol contents. J. Agric. Food Chem..

[58] Giannakopoulou E., Mitsopoulos G., Hagidimitriou M., Papageorgiou V., Komaitis M. (2011). Influence of cultivar, harvesting season and geographical origin on phenolic content in leaves of Greek olive cultivars. Acta Hortic..

[59] Bilgin M., Sahin S. (2013). Effects of geographical origin and extraction methods on total phenolic yield of olive tree (*Olea europaea*) leaves. J. Taiwan Inst. Chem. Eng..

[60] Brahmi F., Mechri B., Dhibi M., Hammami M. (2014). Variation in antioxidant activity and phenolic content in different organs of two Tunisian cultivars of *Olea europaea* L.. Acta Physiol. Plant..

[61] Erel R., Kerem Z., Ben-Gatt A., Dag A., Schwartz A., Zipori I., Basheer L., Yermiyahu U. (2013). Olive (*Olea europaea* L.) tree nitrogen status is a key factor for olive oil quality. J. Agric. Food Chem..

[62] Tekaya M., El-Gharbi S., Mechri B., Chehab H., Bchir A., Chraief I., Ayachi M., Boujnah D., Attia F., Hammami M. (2016). Improving performance of olive trees by the enhancement of key physiological parameters of olive leaves in response to foliar fertilization. Acta Physiol. Plant..

[63] Penel C., Van Cutsem P., Greppin H. (1999). Interactions of a plant peroxidase with oligogalacturonides in the presence of calcium ions. Phytochemistry.

[64] Pasković I., Pecina M., Bronić J., Perica S., Ban D., Goreta Ban S., Poščić F., Palčić I., Herak Ćustić M. (2018). Synthetic zeolite A as zinc and manganese fertilizer in calcareous soil. Commun. Soil Sci. Plant Anal..

[65] Šegota T., Filipčić A. (2003). Köppen’s classification of climates and the problem of corresponding Croatian terminology. Geoadria.

[66] Vuletin Selak G., Perica S., Goreta Ban S., Raduniž M., Poljak M. (2011). Reproductive success after self-pollination and cross-pollination of olive cultivars in Croatia. Hortscience.

[67] Barranco Navero D., Cimato A., Fiorino P., Rallo L., Touzani A., Castañeda C., Serafin F., Trujillo I. (2000). World Catalogue of Olive Varieties.

[68] Marinova D., Ribarova F., Atanassova M. (2005). Total phenolics and total flavonoids in Bulgarian fruits and vegetables. J. Univ. Chem. Technol. Metall..

[69] Miller C.H. (1988). Diurnal temperature cycling influences flowering and node numbers of broccoli. Hortscience.

