# Supplementary material for: Determination of the Variability of Biophenols and Mineral Nutrients in Olive Leaves with Respect to Cultivar, Collection Period and Geographical Location for Their Targeted and Well-Timed Exploitation
Source: Plants (Basel). 2020 Nov 27;9(12):1667. doi: 10.3390/plants9121667 (PMC7760844; doi:10.3390/plants9121667)
Supplement: Supplementary file 1 [file plants-09-01667-s001.pdf]

SUPPLEMENTARY MATERIAL

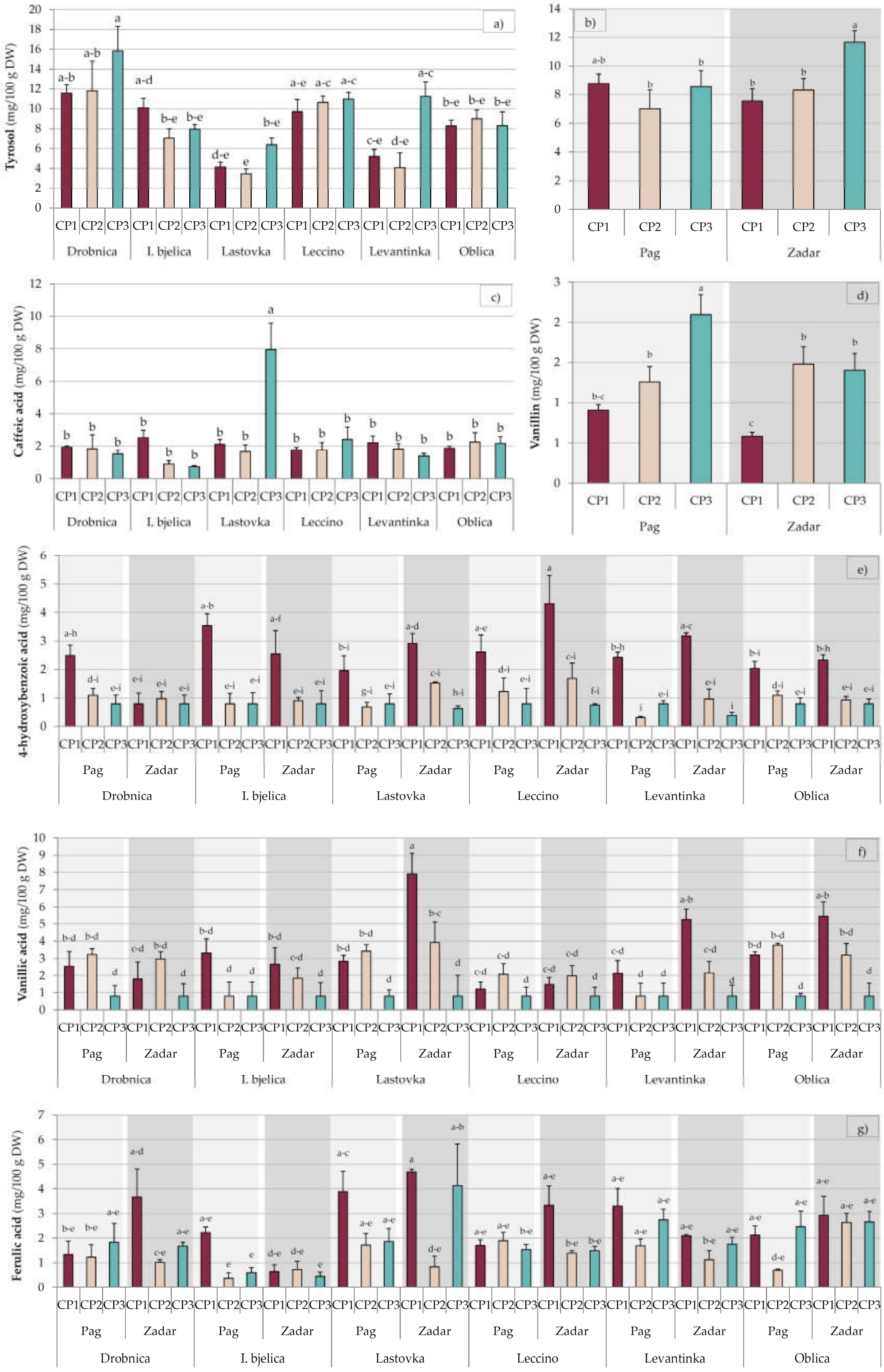

Figure S1. Multiple comparisons of the effects of variety, collection period, and location combinations (highest order interactions) on the concentrations of a) and b) tyrosol, c) caffeic acid, d) vanillin, e) 4-hydroxybenzoic acid, f) vanillic acid, and g) ferulic acid in leaves of Drobnica, Istarska bjelica, Lastovka, Leccino, Levantinka, and Oblica olive varieties collected at different periods (CP1—October 2017, CP2—January 2018, and CP3—March 2018) in two different locations (Pag and Zadar) in Croatia. Different superscript lowercase letters represent statistically significant differences between mean values at $p < 0.05$ obtained by a one-way ANOVA and Tukey's test.

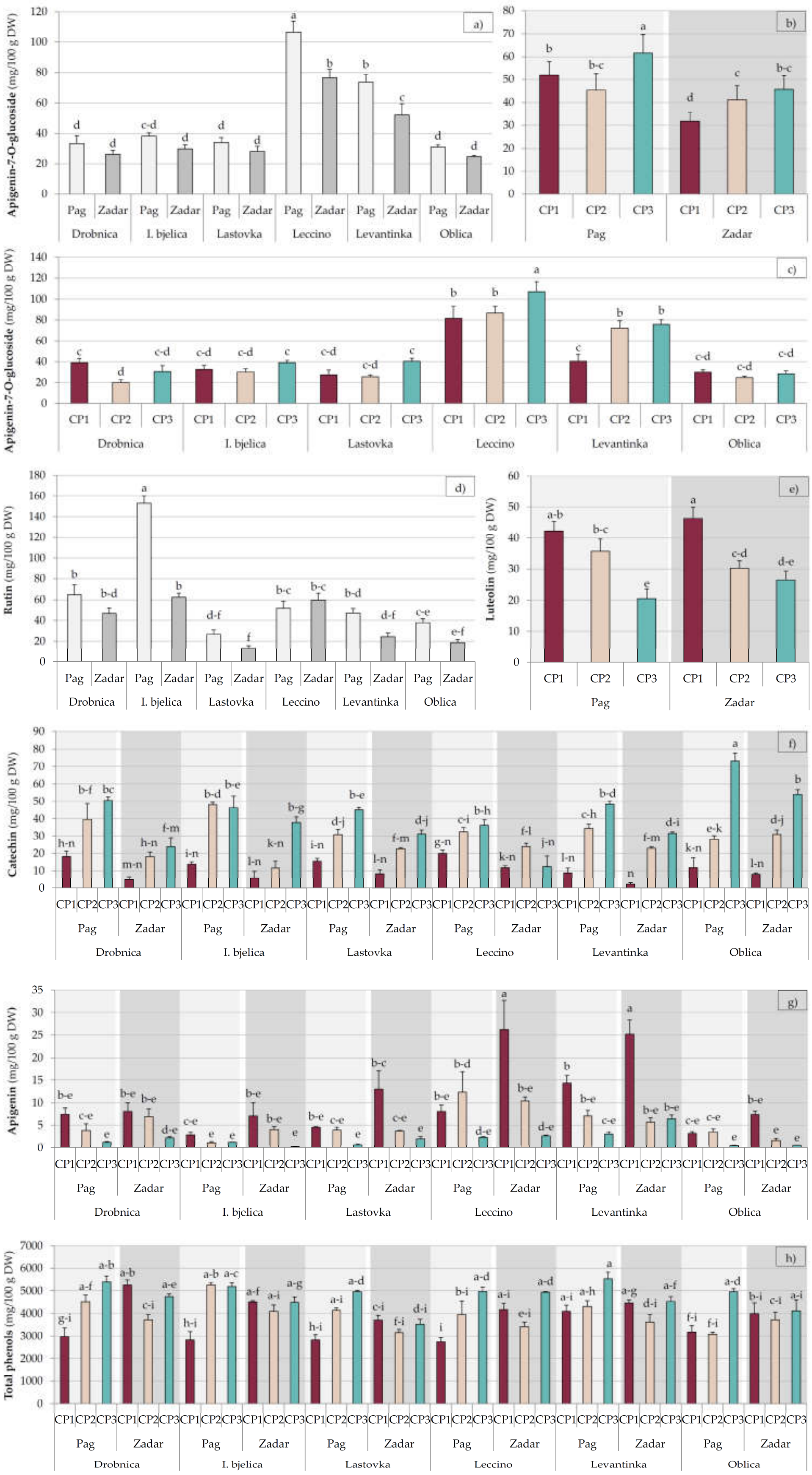

Figure S2. Multiple comparisons of the effects of variety, collection period, and location combinations (highest order interactions) on the concentrations of a) – c) apigenin-7-*O*-glucoside, d) rutin, e) luteolin, f) catechin, g) apigenin, and h) total phenols in leaves of Drobnica, Istarska bjelica, Lastovka, Leccino, Levantinka, and Oblica olive varieties collected at different periods (CP1—October 2017, CP2—January 2018, and CP3—March 2018) in two different locations (Pag and Zadar) in Croatia. Different superscript lowercase letters represent statistically significant differences between mean values at $p < 0.05$ obtained by a one-way ANOVA and Tukey's test.

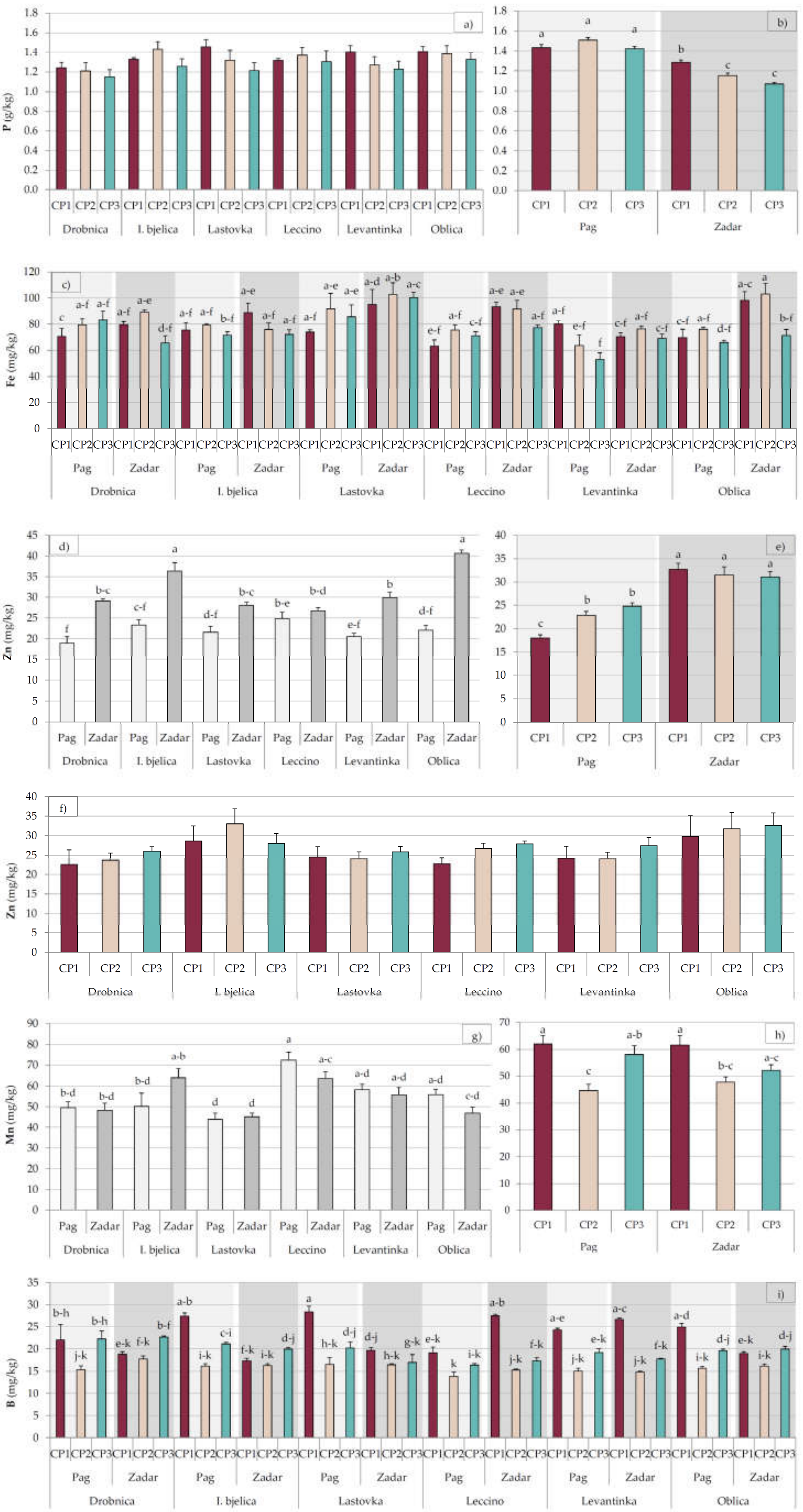

Figure S3. Multiple comparisons of the effects of variety, collection period, and location combinations (highest order interactions) on the concentrations of a) and b) phosphorous (P), c) iron (Fe), d) - f) zinc (Zn), g) and h) manganese (Mn), and i) boron (B) concentrations in leaves of Drobnica, Istarska bjelica, Lastovka, Leccino, Levantinka, and Oblica olive varieties collected at different periods (CP1—October 2017, CP2—January 2018, and CP3—March 2018) in two different locations (Pag and Zadar) in Croatia. Different superscript lowercase letters represent statistically significant differences between mean values at $p < 0.05$ obtained by a one-way ANOVA and Tukey's test.

Table S1. Chemical properties of the Calcocambisol soil at two locations (Novalja, Island of Pag 44°32′53′′ N and Poličnik near Zadar 14°52′58′′E) used in the study

Parameter	Pag	Zadar
pH (H_2O)	7.89	7.91
pH (KCl)	7.22	7.09
Total N (%)	0.41	0.19
P (mg/100g)	7.07	3.41
K (mg/100g)	52.95	23.65
Organic matter (%)	6.90	3.11

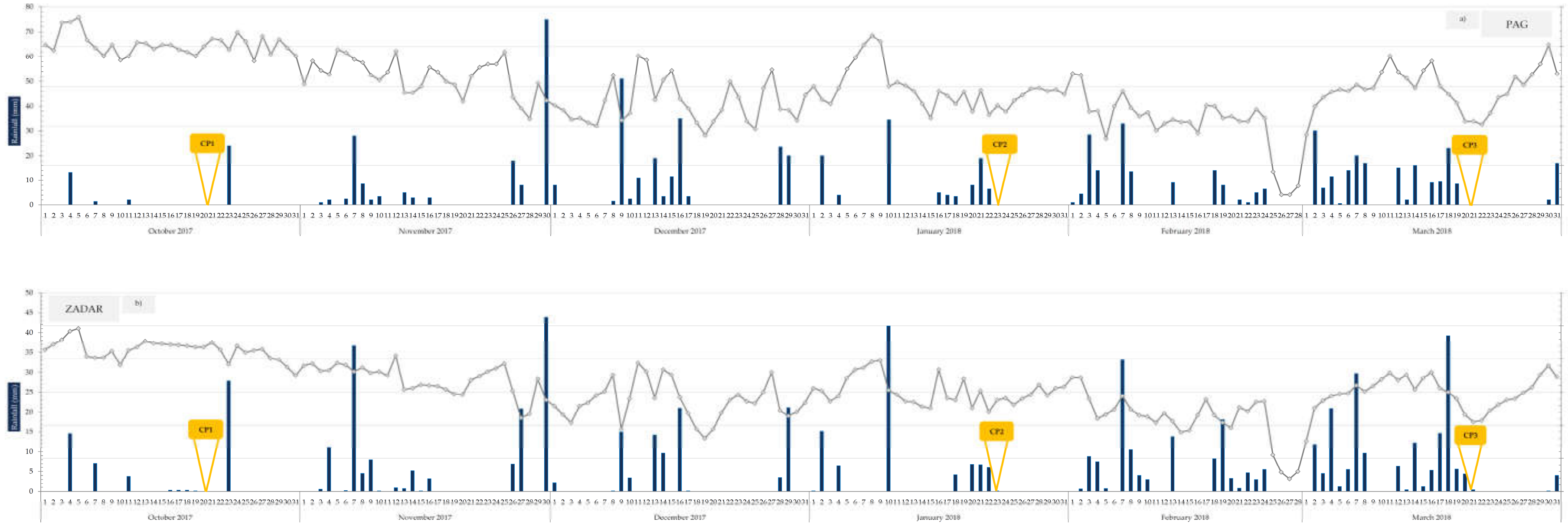

Figure S4. Average daily rainfall (mm) and temperatures (°C) measured from the beginning of October 2017 until the end of March 2018 on the experiment locations a) Pag and b) Zadar with indicated olive leaf collecting periods (CP1 – CP3).

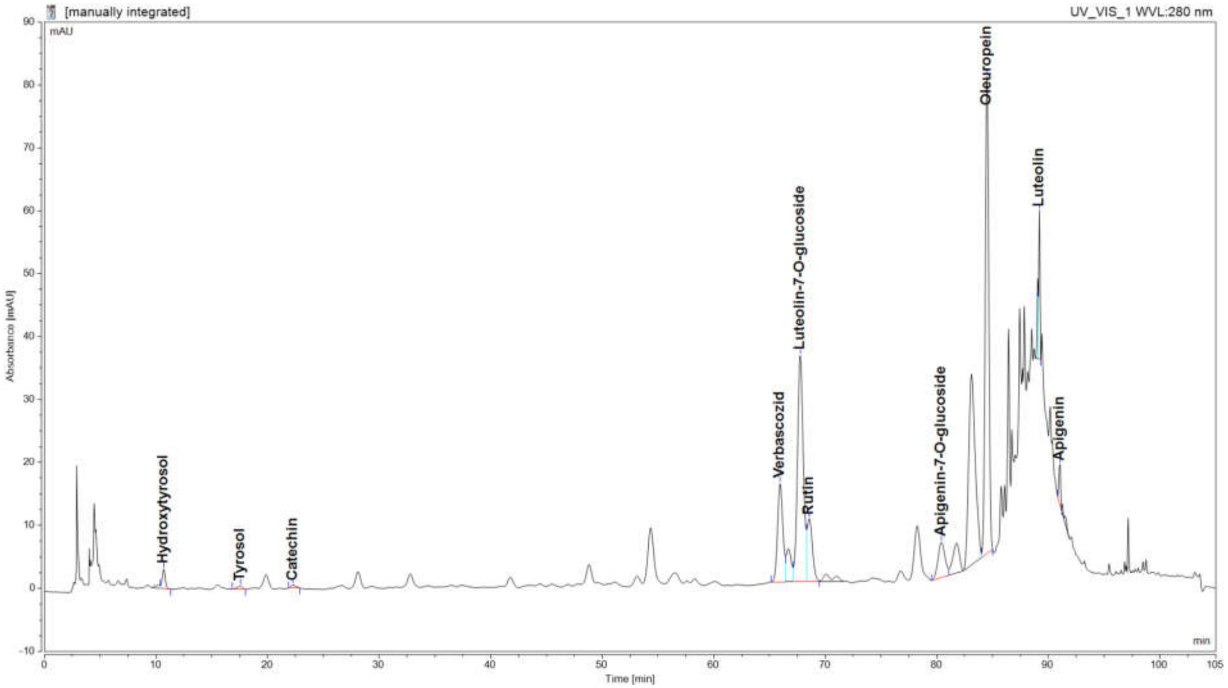

Figure S5. Example of an olive leaf extract HPLC chromatogram recorded at 280 nm used for identification and quantification of hydroxytyrosol, tyrosol, vanillin, apigenin-7-*O*-glucoside, and catechin.
